# Evaluation of Five Screening Tools in Detecting Physical Frailty in Cirrhosis and Their Prognostic Role

**DOI:** 10.3390/jcm13175169

**Published:** 2024-08-30

**Authors:** Eleni Geladari, Theodoros Alexopoulos, Larisa Vasilieva, Roxane Tenta, Iliana Mani, Vassilios Sevastianos, Alexandra Alexopoulou

**Affiliations:** 1 3rd Department of Internal Medicine and Liver Outpatient Clinic, Evangelismos General Hospital, 10676 Athens, Greece; elgeladari@gmail.com (E.G.); vsevastianos@gmail.com (V.S.); 2 Gastroenterology Department, Medical School, Laiko General Hospital, National & Kapodistrian University of Athens, 11527 Athens, Greece; theoalex@windowslive.com; 3 Department of Gastroenterology, Alexandra General Hospital, 11528 Athens, Greece; larisatheo@yahoo.gr; 4 Department of Nutrition & Dietetics, School of Health Sciences and Education, Harokopio University of Athens, 17676 Athens, Greece; rtenta@hua.gr; 5 2nd Department of Internal Medicine and Research Laboratory, Medical School, Hippokration General Hospital, National and Kapodistrian University of Athens, 11527 Athens, Greece; ilianamani@windowslive.com

**Keywords:** physical frailty, Liver Frailty Index, Short Physical Performance Battery, Fried frailty phenotype, Clinical Frailty Scale, 6-Minute Walk Test, Cohen’s kappa measurement of agreement, prognosis

## Abstract

**Background**: Physical frailty (PF) is a syndrome of decreased physical function and reserves, preventing patients from coping with stressful events. PF screening tools in patients with liver cirrhosis (LC) can help evaluate the risk of complications and death. The aim of this study was to assess the performance of five screening tools in detecting PF and their ability to predict 18-month mortality in LC. **Methods**: The Short Physical Performance Battery (SPPB), Fried frailty phenotype (FFP), Clinical Frailty Scale (CFS) and 6-Minute Walk Test (6MWT) were compared with the Liver Frailty Index (LFI) as the method of reference. Patients with an LFI ≥ 4.5, SPPB ≤ 8, FFP ≥ 3, CFS ≥ 6 points, and those walking <250 m, were considered frail. **Results:** A total of 109 consecutive patients with stable LC were included [63.3% male, median age 62 years, (IQR 52–70), MELD 9 (7–14.5), 46.8% with decompensated LC (DC)]. PF was present in 23.9%, 27.5%, 41.3%, 13.8%, and 28.4% as assessed by the LFI, SPPB, FFP, CFS, and 6MWT, respectively. Cohen’s kappa measurement of agreement of four of the tools with LFI was 0.568, 0.334, 0.439, and 0.502, respectively (*p* < 0.001 for each). Kaplan–Meier survival curves at 18 months showed higher mortality in frail patients compared to non-frail patients by any method (log rank *p* < 0.05). In the multivariate models, PF defined by any method emerged as an independent prognostic factor of 18-month mortality after adjustment for age, gender, and MELD-score. **Conclusions**: Patients characterized as frail by five screening tools were not identical. However, PF defined by either method was proven to be an independent poor prognostic factor for long-term mortality after adjustment for covariates.

## 1. Introduction

Global frailty is a multidimensional syndrome of decreased physiologic reserves, functional status impairment, and increased vulnerability to stress factors [1]. It includes physical function decline as a result of loss of skeletal muscle and function (physical frailty (PF)) [2], cognitive, and psychological derangements [3]. PF can be considered as pre-disability, with disability defined as needing assistance with the basic activities of daily living (ADL) [2].

PF and sarcopenia [4,5] or disability [6] are often interrelated and it is common that individuals with PF have disability and/or sarcopenia and vice versa [2]. However, they are also clinically and conceptually distinct and have different diagnostic criteria and screening tools [2,4,6,7]. In addition, interventions to prevent and/or to manage frailty, sarcopenia, or disability may differ [2,3,4,8].

PF was first recognized in the field of geriatrics [9] and then in patients with systemic deficiencies (cardiovascular, neurologic, renal, etc.) [10,11,12]. In the setting of cirrhosis, PF was mainly studied in decompensated cirrhosis [13]. Previous studies have shown that PF was an independent predictor of mortality in patients with decompensated cirrhosis in transplant wait-list or in post-transplant setting [13,14,15].

Multiple tools have been used to evaluate the presence of PF including the Liver Frailty Index (LFI), Short Physical Performance Battery (SPPB), Fried frailty phenotype (FFP), the Clinical Frailty Scale (CFS), 6-Minute Walk Test (6MWT), short gait speed or grip strength (single measure), Karnofsky Performance Status (KPS), etc. [2,3,7]. However, most of them were studied in the elderly population and their diagnostic and prognostic performance in cirrhosis were not well documented.

The aim of the present study was to assess the effectiveness of four screening tools in detecting PF (SPPB, FFP, 6MWT, and CFS) by comparing them with a method that is specifically designed for patients with liver disease and is considered as one of the most studied PF tools, that is the LFI, in stable cirrhotic patients with either compensated or decompensated cirrhosis. The ability of the PF tools to predict 18-month mortality was also calculated.

## 2. Materials and Methods

### 2.1. Study Sample

This prospective study was conducted from March 2021 to March 2024. Patients with liver cirrhosis from the outpatient clinic or the wards of the 2nd Department of Internal Medicine, Hippokration General Hospital and the 3rd Department of Internal Medicine, Evangelismos General Hospital, were included. Hospitalized patients were enrolled just before hospital discharge while they were in a stable condition without acute clinical events or hepatic encephalopathy. Patients with alcoholic cirrhosis were included if they have ceased alcohol intake at least one month before enrolment. The diagnosis of cirrhosis was based on liver histology and/or a combination of clinical, laboratory, imaging, and endoscopic data. Cirrhosis was considered as decompensated in patients with history of ascites, variceal bleeding, hepatic encephalopathy, or jaundice of non-obstructive cause (bilirubin >3 mg/dL for non-cholestatic and >10 mg/dL for cholestatic causes of cirrhosis). Patients with primary liver cancer or extrahepatic malignancies, liver transplantation, immunosuppressive therapy other than corticosteroids, human immunodeficiency virus infection and cardiac, renal, or respiratory failure were excluded from this study. A control group of age- and sex-matched patients was also recruited. Every participant was informed about the aim of this study and signed an informed consent form. Only patients who could undergo the physical frailty assessment methods were included in this study. The protocol was approved by the Hospital Scientific Committee.

### 2.2. Clinical Data

Clinical manifestations relating to cirrhosis and laboratory parameters (including biochemical and clotting profile) were prospectively recorded. MELD and Child–Pugh scores were calculated to evaluate the severity of liver disease.

Patients were prospectively followed up during hospitalization and if discharged, at the outpatient clinic, using electronic medical records and monthly telephone calls during the 18-month follow-up period.

### 2.3. Anthropometric Measurements

Body weight (BW) was measured using an electronic scale and standing height was measured using a stadiometer (Seca 769 digital scale and Seca 220 stadiometer, respectively; Seca Medical Systems, Hamburg, Germany). Dry body weight was calculated by subtracting 5% of the computed BW for mild ascites, 10% for moderate ascites, and 15% for tense ascites, with an additional 5% subtracted if bilateral pedal edema was present, as suggested by European Association for the Study of the Liver [1]. Dry body mass index (BMI) (kg/m^2^) and history of falls over the past 2 years were also determined.

Muscle strength for all included patients and controls was measured using a calibrated hydraulic hand dynamometer (Jamar Hydraulic Dynamometer, model 5030j1; Jamar Co., Duluth, MN, USA). Three handgrip measurements from each hand were recorded for each participant [16] and maximum measurements were used for statistical analysis.

### 2.4. Sarcopenia Diagnosis

According to the updated EWGSOP-2 criteria [4], sarcopenia is diagnosed when low muscle strength and decreased muscle mass and/or quality (myosteatosis), are present. In the current study, low SMI cut-off values (<50 cm^2^/m^2^ for men and <39 cm^2^/m^2^ for women) set by Carey et al. [17] were used to identify patients with decreased muscle mass based on CT scans. Patients with low muscle strength, accompanied by decreased muscle mass and/or myosteatosis, were classified as sarcopenic.

### 2.5. Physical Frailty Tools

Five physical frailty screening tools were assessed for their effectiveness in detecting physical frailty.

LFI: Designed specifically for patients with liver disease [13], it includes three components: time to do five chair stands, seconds holding a three-position balance, and dominant handgrip strength assessments. In this study, frail (≥4.5) vs. non-frail (0–4.4) persons were compared.

SPPB: Comprising three components: time to complete a 2.4, 3, or 4 m walk at the participant’s usual pace, time to rise from a chair five times, and the ability to stand for up to 10 s with feet positioned in each of three ways (side-by-side, semi-tandem, and tandem) [18]. Scores range from 0 (worst) to 12 (best) with a total score of ≤8 points indicating frailty.

FFP: Uses five criteria: self-reported weight loss, exhaustion using CES-D scale [19], physical activity measured by the Physical Activity Scale for the Elderly (PASE) with a cutoff of 90 for low activity [20], walk speed, and grip strength. An individual is classified as frail if ≥3 criteria are met.

6MWT: A walking distance cut-off of <250 m defines PF [21].

CFS: Comprising nine components, classifies individuals from very fit (1) to terminally ill (9) [22]. In this study, frail individuals were those with moderate (6), severe (7), or very severe frailty (8). Terminally ill individuals (9) were not included.

### 2.6. Statistical Analysis

All data were analyzed using SPSS (version 23.0; SPSS Inc., Chicago, IL, USA). Quantitative variables were expressed as median values and interquartile ranges (IQR), and categorical variables as counts and percentages. The Mann–Whitney U test was used for comparisons of continuous variables between groups, and the chi-squared test was used for categorical variables. A two-tailed *p*-value less than 0.05 was considered statistically significant.

Cohen’s kappa (K) statistical measurement was used to quantify the level of agreement between two screening tools. The kappa result was interpreted as follows: values ≤0 indicating no agreement, 0.01–0.20 as none to slight, 0.21–0.40 as fair, 0.41–0.60 as moderate, 0.61–0.80 as substantial, and 0.81–1.00 as almost perfect agreement.

Sensitivity, specificity, positive and negative predictive values were measured using the MedCalc Diagnostic test evaluation calculator. The prognostic performance of LFI was evaluated using the area under the receiver operating characteristic (AUROC) curve, represented by the concordance (c)-statistic. Prognostic accuracy was considered good if c-statistic was greater than 0.70. The cut-off value of LFI in predicting mortality was chosen based on the optimal combination of sensitivity and specificity. This optimal cutoff value for LFI was then used in Kaplan–Meier curve and Cox regression model. ROC curves were compared using MedCalc statistical software (version 22.032; MedCalc Software Ltd., Ostend, Belgium).

Cumulative probabilities of death during follow-up were calculated using the Kaplan–Meier method and compared between groups using log-rank test. The Cox proportional hazards regression model was employed to estimate risk factors that were associated with poor prognosis. Factors with a p value of less than 0.05 in the univariate analysis, along with age, gender, and MELD score, were included in the multivariate model. Non-significant factors were removed through a backward selection process.

## 3. Results

### 3.1. Patient Characteristics

One hundred and nine (109) consecutive patients with stable liver cirrhosis (without acute events) were included in this study [63.3% male, median age 62 years, (IQR 52–70), MELD 9 (7–14.5), 46.8% with decompensated cirrhosis (DC)]. The causes of liver cirrhosis were 41.3% alcohol related, 22% viral, and 36.7% miscellaneous (Table 1). Frail patients (diagnosed by LFI) were older (*p* < 0.001), more sarcopenic (*p* < 0.001), had more severe liver disease [higher MELD score (*p* = 0.024), more frequent hepatic encephalopathy (*p* = 0.020)], and higher serum creatinine (*p* = 0.003). 

### 3.2. Prevalence of Physical Frailty According to the Screening Method Applied

PF was present in 23.9%, 27.5%, 41.3%, 13.8%, and 28.4% of patients as assessed by the LFI, SPPB, FFP, CFS, and 6MWT, respectively. Frailty was more prevalent in patients with decompensated cirrhosis compared to those with compensated cirrhosis, reaching statistical significance when PF was diagnosed by FFP (*p* < 0.001) or 6MWT (*p* < 0.001). In the control group, 6.3%, 6.3%, 15.6%, 6.3%, and 9.4% were frail according to the LFI, SPPB, FFP, CFS, and 6MWT, respectively. PF was less frequently present in controls compared to patients with cirrhosis, by all screening tools except for the CFS (Table 2).

### 3.3. Diagnostic Evaluation of Each Screening Method Compared to LFI as the Reference Method

SPPB showed a moderate (Cohen’s kappa measurement 0.568), FFP a fair (K = 0.334), CFS a moderate (K = 0.439), and 6MWT a moderate agreement (K = 0.502) with the LFI (*p* < 0.001, for all tests) (Table 3). Using SPPB as the reference tool then, the 6-Minute Walk Test displayed the maximum agreement (substantial) with SPPB (K = 0.659) (*p* < 0.001) (Supplementary Table S1).

SPPB, CFS, and 6MWT showed the highest diagnostic accuracy (effectiveness) and FFP demonstrated the lowest diagnostic accuracy with LFI (Table 4).

### 3.4. Survival

A new cutoff value for LFI was calculated as the 4.5 cutoff value and was not proved a good predictor of 18-month mortality in our cohort. Based on the ROC curve, the optimal cutoff of LFI to differentiate survivors from non-survivors at 18 months was 4.34, offering a sensitivity of 70% and specificity of 71.8% (AUROC: 0.729, *p* = 0.002) (Supplementary Figure S1). The ROC curves of the remaining PF screening tools as well as the pairwise comparison between them are presented in the Supplementary Materials (Supplementary Table S2 and Table S3, Supplementary Figure S2).

**Figure 1:**
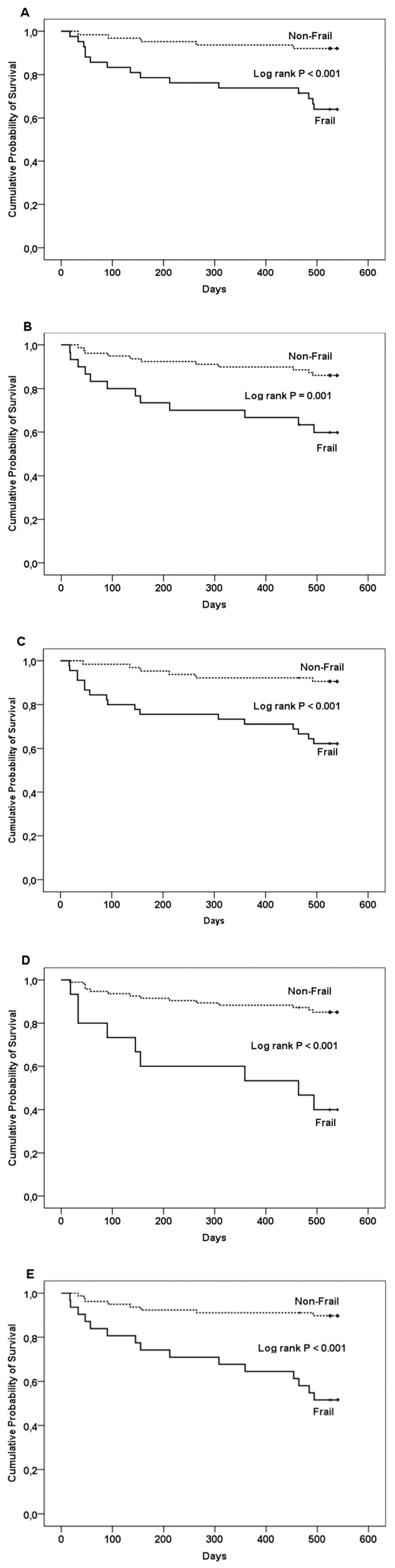
Kaplan–Meier survival curves for frail vs. non-frail patients with 5 screening tools for frailty. (**A**) Liver Frailty Index; (**B**) Short Physical Performance Battery; (**C**) Fried frailty phenotype; (**D**) Clinical Frailty Scale; (**E**) 6-Minute Walk Test.

At 18 months, 21 patients (41.2%) with decompensated cirrhosis and 2 with compensated cirrhosis (3.4%) had died. Patients with PF displayed a higher mortality rate compared to those without at 18 months (log rank *p* < 0.001 for LFI, log rank *p* = 0.001 for SPPB, log rank *p* < 0.001 for FFP, log rank *p* < 0.001 for CFS, and log rank *p* < 0.001 for 6MWT) (Figure 1A–E). 

Cox univariate analysis for age, gender, MELD score, and LFI, SPPB, FFP, CSF, and 6MWT showed that the MELD score (*p* < 0.001) and PF diagnosed by LFI (*p* = 0.001), SPPB (*p* = 0.003), FFP (*p* = 0.001), CFS (*p* < 0.001), and 6MWT (*p* < 0.001) were associated with mortality at 18 months (Table 5). 

Five multivariate analysis models were created, adjusting PF diagnosed by any one of the five PF screening tools for age, gender, and MELD score (dichotomized as ≥15 and <15). PF tested by LFI (HR 3.679, 95%CI 1.303-10.385, *p* = 0.014), by SPPB (HR 2.735, 95%CI 1.189–6.290, *p* = 0.018), by FFP (HR 3.417, 95%CI 1.329–8.783, *p* = 0.011), by CFS (HR 2.479, 95%CI 1.015–6.057, *p* = 0.046), and by 6MWT (HR 3.631, 95%CI 1.480–8.906, *p* = 0.005) emerged as independent prognostic factors of mortality (for LFI, the 4.34 cutoff was used and for the rest, the cutoffs did not change) (Table 5).

Similarly, Kaplan–Meier survival curves at 12 months showed that patients with PF displayed an increased mortality rate compared to those without (log rank *p* = 0.004 for LFI, log rank *p* = 0.002 for SPPB, log rank *p* = 0.003 for FFP, log rank *p* < 0.001 for CFS and log rank *p* = 0.001 for 6MWT) (Supplementary Table S4). Furthermore, significantly increased mortality rates in frail persons were demonstrated by all screening tools at 3 and 6 months (Supplementary Table S5).

## 4. Discussion

In a cohort of individuals with compensated and decompensated cirrhosis, the prevalence of PF varied depending on the screening method employed. Our findings indicate that PF was associated with sarcopenia and more advanced liver disease. All four methods utilized demonstrated moderate to substantial agreement with the LFI, which is the best validated screening tool of assessing PF in liver cirrhosis [13,14,15]. Among the methods, those using objective criteria to determine PF exhibited the strongest agreement. PF assessed by each of the five methods emerged as an independent predictor of long-term mortality after adjusting for covariates.

According to the existing literature, the prevalence of PF in patients with liver cirrhosis ranges from 18% to 43% contingent on the assessment tool and the disease severity [3,5]. Unlike a previous investigation, where the prevalence of PF was similar across the five screening methods [23], in the current study, PF ranged from 13.8% using the CFS to 41.3% using the FFP. The prevalence was somewhat similar (about 25%) using LFI, SPPB, and 6MWT. This variability is attributable to differences in measurement methods, comprising either objective or subjective criteria. For instance, tools like LFI, SPPB, and 6MWT, which incorporate objective physical performance criteria, exhibited moderate or substantial agreement between themselves and with LFI. Notably, the most significant agreement was observed between LFI and SPPB, likely due to the shared physical performance criteria. Conversely, FFP, which includes patient-filled questionnaires along with objective criteria, showed the highest PF prevalence but the lowest agreement with LFI. Taking into account that FFP was the most time-consuming method, it was deemed as the least convenient PF tool in our study.

CFS, another subjective tool reliant on health-care provider estimation, demonstrated better agreement with LFI when the cutoff used was 6 or higher (indicating moderate frailty and more). In contrast, when a cutoff of 5 or higher (including mild frailty) was applied, the prevalence of frailty resembled that measured by LFI, SPPB, and 6MWT, albeit with poor agreement between CFS and LFI. Consequently, we opted for a cutoff of 6 or higher. Nonetheless, as CFS grading depends on healthcare provider opinion, it may suffer from overestimation or underestimation of a patient’s physical condition. Thus, we considered that while the agreement between tools using objective criteria was deemed adequate in our study, the agreement between tools using objective and those using subjective criteria was poor.

Consistent with prior research [13,14,15], we observed that frail individuals had more severe liver disease (as assessed by MELD and hepatic encephalopathy) and comorbidities such as renal impairment. PF was more prevalent in decompensated versus compensated cirrhosis across all tools, with statistically significant differences noted in two of them. The prevalence of frailty was higher in patients with cirrhosis than in controls, indicating that cirrhosis was associated with PF. Various factors like hypercatabolic state, systemic inflammation, ascites and its consequences, which worsen as the disease progresses, may contribute to frailty, leading to reduced physical activity and increased vulnerability to stress [3,5].

Muscle loss is considered one of the major drivers of PF [5,24], so frail persons in the current study were more frequently sarcopenic. Sarcopenia denoting a loss of skeletal muscle mass and function is interrelated to physical frailty [3,5]. Notably, handgrip strength, a component of LFI, is a key element of sarcopenia.

PF emerged as an independent predictor of mortality across all screening tools used, reflecting poor functional status and reserves in frail patients who were older, more frequently sarcopenic and had more advanced liver disease. Unlike previous studies focused on liver transplant waitlist patients, our study included a mixed population of compensated and decompensated patients and extended the follow-up period to 18 months, revealing PF as a robust predictor of mortality over this period. Hence, we found that PF determined by the standard cut-offs for SPPB, 6MWT, CFS, and FFP performed well as an independent predictor of mortality not only at 12 but also at 18 months. Short- or medium-term mortality prediction was commonly examined in the literature by all aforementioned tools with good results [13,23,25,26,27], but only scarcely more than 12 months prediction was explored [28]. Regarding LFI, despite the 4.5 cut-off optimized agreement between PF methods, it was not ideal for mortality prediction. Thus, we proposed a new cut-off of 4.34, which effectively predicted mortality after adjusting for age, gender, and severity of liver disease. The LFI cutoff values for mortality prediction varied widely in the previous studies, ranging from 3.7 to 5.2 due to population differences and observation periods [29]. The wide variation in LFI cut-off values for mortality prediction in previous studies may also reflect the wide distribution of possible measurements, a unique feature for LFI. Moreover, it was reported that 50% of LT candidates exhibited a worsening of LFI during time on the waiting list [30] and consequently the cutoff values for the prediction of death may be modified [30].

Our study is important because it investigated the implementation of PF screening methods with the commonly used cut-offs in a cohort of both compensated and compensated cirrhosis from two tertiary centers during a relatively long period of follow-up. In addition, we explored the concordance among tools for the identification of frail persons. It should be noted that only one study in the past compared the different PF scores but used a different cohort and methodology [23].

Furthermore, we only enrolled patients in a stable condition from the outpatient clinic or before discharge from the hospital attempting to avoid over-estimation of PF score due to acute complications of cirrhosis or cognitive impairment. This may be considered as one of the strengths of this study.

This study also acknowledges limitations. First, the number of the included patients is modest. Secondly, PF tool measurements were conducted by a single clinician, albeit experienced.

In conclusion, we demonstrated that PF prevalence varied depending on the screening tool used. Three of the screening tools and particularly those which used objective criteria were concordant, simple to apply, and exhibited a similar prevalence of physical frailty. All five screening tools were predictive of clinical outcome after adjusting for severity of liver disease. Most of the cut-offs used in the literature to determine frailty functioned well in the differentiation of survivors and non-survivors even in this population of both compensated and decompensated cirrhotic patients.

## Figures and Tables

**Table 1:** Baseline characteristics of the total study sample and by frailty state (Liver Frailty Index) (median, interquartile range, or percentages).

Characteristic	Total	Frail (N = 26)	Non Frail (N = 83)	*p* Value
Gender (Male)	69 (63.3%)	18 (69.2)	51 (61.4)	0.472
Age (years)	62 (52–70)	70.5 (63.7–78.5)	60 (50–67)	<0.001
Dry BMI (kg/m^2^)	26.8 (24.7–29.9)	26.5 (23.6–30.6)	26.9 (25–29.1)	0.792
Etiology of cirrhosis • Viral (N%) • Alcoholic (N%) • Other (N%)	45 (41.3) 24 (22) 40 (36.7)	8 (30.8) 5 (19.2) 13 (50)	37 (44.6) 19 (22.9) 27 (32.5)	0.262
Child–Pugh Score (points)	6 (5–8)	7 (5–8)	6 (5–8)	0.327
Sarcopenia (N%)	56 (51.4%)	25 (96.2%)	31 (37.3%)	<0.001
Skeletal Muscle Index (cm^2^/m^2)^	46.8 (42.7–54.0)	42.7 (34.3047.8)	48.9 (43.5–54.2)	0.011
MELD score	9 (7–14.5)	11 (8.2–17.2)	9 (7–13)	0.024
History of hepatic encephalopathy (N%)	19 (17.4%)	8 (30.8%)	11 (13.3%)	0.020
History of falls (N%)	49 (45%)	15 (57.7%)	34 (41%)	0.069
Albumin (g/dL)	4 (3.4–4.4)	3.8 (3.2–4.4)	4 (3.4–4.4)	0.327
INR	1.1 (1.0–1.3)	1.2 (1.1–1.4)	1.1 (1.0–1.3)	0.211
Creatinine (mg/dL)	0.8 (0.7–1.1)	1.1 (0.8–1.4)	0.8 (0.7–1)	0.003
Total Bilirubin (mg/dL)	1.1 (0.7–1.8)	1.2 (0.8–1.6)	1 (0.6–2)	0.572
Ascites (N%)	57 (52.3%)	16 (61.5%)	41 (49.4%)	0.089
Variceal bleeding (N%)	25 (22.9%)	6 (23.1%)	19 (22.9%)	0.198

INR, International Normalized Ratio; MELD, Model for End-Stage Liver Disease; Median (Interquartile Range) is used for continuous variables; individuals with LFI ≥ 4.5 were considered as frail; no patients, Child Pugh A = 58, B = 41, C = 10.

**Table 2:** Prevalence of frail persons according to the state of cirrhosis (compensated or decompensated) and in the control group.

Physical Frailty Tool	Total	Compensated Cirrhosis (N = 58)	Decompensated Cirrhosis (N = 51)	*p*_1_ Value	Controls (N = 32)	*p*_2_ Value
Liver Frailty Index	26 (23.9%)	11 (19%)	15 (29.4%)	0.442	2 (6.3%)	0.028
Short Physical Performance Battery	30 (27.5%)	13 (22.4%)	17 (33.3%)	0.444	2 (6.3%)	0.012
Fried Frailty Phenotype	45 (41.3%)	15 (25.9%)	30 (58.8%)	<0.001	5 (15.6%)	0.008
Clinical Frailty Scale	15 (13.8%)	4 (6.9%)	11 (21.6%)	0.085	2 (6.3%)	0.251
6-Minute Walk Test	31 (28.4%)	8 (13.8%)	23 (45.1%)	<0.001	3 (9.4%)	0.027

*p*_1_, comparison between compensated and decompensated cirrhosis; *p*_2_, comparison between patients and controls; individuals with LFI ≥ 4.5 were considered as frail.

**Table 3:** Measurement of agreement of each of the 4 PF screening tools with Liver Frailty Index.

Physical Frailty Tool	Prevalence	Agreement between Methods in the Diagnosis of Frail Persons	Agreement between Methods in the Diagnosis of Non-Frail Persons	Measurement of Cohen’s Kappa Agreement	*p* Value
Short Physical Performance Battery	30 (27.5%)	19 (73.1%)	72 (86.7%)	0.568	<0.001
Fried Frailty Phenotype	45 (41.3%)	19 (73.1%)	57 (68.7%)	0.334	<0.001
Clinical Frailty Scale	15 (13.8%)	11 (42.3%)	78 (95.2%)	0.439	<0.001
6-Minute Walk Test	31 (28.4%)	18 (71.6%)	70 (84.3%)	0.502	<0.001

Individuals with LFI ≥ 4.5 were considered as frail.

**Table 4:** Diagnostic evaluation of the 4 physical frailty screening tools compared to Liver Frailty Index.

Physical Frailty Tool	Sensitivity (%)	Specificity (%)	Positive Predictive Value (%)	Negative Predictive Value (%)	Accuracy (%)
Short Physical Performance Battery	73.08	86.75	63.33	91.14	83.5
Fried Frailty Phenotype	71.4	73.4	40	91.2	73.1
Clinical Frailty Scale	42.31	95.18	73.33	84.04	82.6
6-Minute Walk Test	58.06	89.7	69.23	84.34	80.7

Individuals with LFI ≥ 4.5 were considered as frail.

**Table 5:** Estimated hazard ratio for 18-month mortality of patients diagnosed with physical frailty in univariate analysis and after adjustment for age, gender, and MELD score.

	Univariate Analysis	*p* Value	Multivariate Analysis	*p* Value
	HR (95% CI)		HR (95% CI)	
Age (per year)	1.027 (0.991–1.063)	0.142		
Gender (Male)	0.891 (0.386–2.059)	0.788		
MELD score	8.262 (3.485–19.584)	<0.001	6.230 (2.421–16.032) * 7.035 (2.929–16.898) ** 6.729 (2.807–16.133) *** 6.361 (2.541–15.924) **** 5.698 (2.317–14.009) *****	<0.001 <0.001 <0.001 <0.001 <0.001
Liver Frailty Index *	5.295 (1.922–14.582)	0.001	3.679 (1.303–10.385)	0.014
Short Physical Performance Battery **	3.474 (1.531–7.882)	0.003	2.735 (1.189–6.290)	0.018
Fried Frailty Phenotype ***	4.824 (1.900–12.246)	0.001	3.417 (1.329–8.783)	0.011
Clinical Frailty Scale ****	5.437 (2.345–12.603)	<0.001	2.479 (1.015 –6.057)	0.046
6-Minute Walk Test *****	5.810 (2.458–13.731)	<0.001	3.631 (1.480–8.906)	0.005

Each PF tool was adjusted for age, gender and MELD score. Each time only one of the PF tools (LFI *, SPPB **, FFP ***, CSF **** and 6MWT *****), entered in the equation, five multivariate models were created, each one corresponds to asterisks (1–5 asterisks). MELD score was also an independent prognostic factor of survival and the values of HR (95%CI) for each of the multivariate models separately are noted; individuals with LFI ≥ 4.34 are considered as frail; only the statistically significant variables are shown in multivariate analysis columns.

## Data Availability

Data are included in the article. Further inquiries can be directed to the corresponding author.

## Supplementary Materials

The following supporting information can be downloaded at: https://www.mdpi.com/article/10.3390/jcm13175169/s1, Table S1: Measurement of agreement of each of the 4 PF screening tools with SPPB; Table S2: Area under the curve of the ROC curves of the 5 physical frailty tools for the prediction of 18 months mortality; Table S3: Pairwise comparison of ROC curves between the 5 physical frailty tools for the prediction of 18 months mortality; Table S4: Estimated hazard ratio for 12-month mortality of patients diagnosed with physical frailty in univariate analysis and after adjustment for age, gender, and MELD score; Table S5: Log Rank tests from Kaplan Meier survival curves for frail versus non-frail patients with 5 screening tools for frailty; Figure S1: Receiver operating characteristic (ROC) curve of the Liver Frailty Index measurement for differentiating survivors from non-survivors. The cut-off point of 4.34 yielded a sensitivity of 70% and specificity of 71.8%; Figure S2: Comparison of predictive ability of the AUROCs corresponding to Liver Frailty Index, Short Physical Performance Battery, Fried Frailty Phenotype, Clinical Frailty Scale, and 6-Min Walk Test for 18-month mortality.
