# Supplementary material for: Evaluation of Five Screening Tools in Detecting Physical Frailty in Cirrhosis and Their Prognostic Role
Source: J Clin Med. 2024 Aug 30;13(17):5169. doi: 10.3390/jcm13175169 (PMC11396431; doi:10.3390/jcm13175169)

Supplemantary Table S1. Measurement of agreement of each of the 4 PF screening tools with SPPB

Physical Frailty tool	Measurement of agreement K cohen	p value
Liver Frailty Index	0.568	<0.001
Fried Phenotype	0.383	<0.001
Clinical Frailty-Scale	0.537	<0.001
6-min Walk Test	0.659	<0.001

Individuals with LFI ≥4.5 were considered as Frail

Supplementary Table S2. Area under the curve of the ROC curves of the 5 physical frailty tools for the prediction of 18 months mortality

Variable	AUC	Standard error	95% CI
LFI	0.729	0.053	0.633 to 0.811
SPPB	0.736	0.054	0.641 to 0.818
FFP	0.751	0.050	0.657 to 0.830
CFS	0.834	0.041	0.749 to 0.900
6-min-WT	0.780	0.055	0.689 to 0.855

LFI, Liver Frailty Index; SPPB, Short Physical Performance-Battery; FFP, Fried phenotype; CFS, Clinical Frailty-Scale; 6-min-WT 6-min walk test

Supplementary Table S3. Pairwise comparison of ROC curves between the 5 physical frailty tools for the prediction of 18 months mortality

		Difference between areas	Standard error	P value
LFI compared to	SPPB	0.008	0.045	0.882
	FFP	0.022	0.057	0.698
	CFS	0.106	0.049	0.032
	6-min-WT	0.518	0.053	0.331
SPPB compared to	FFP	0.014	0.051	0.785
	CFS	0.097	0.036	0.006
	6-min-WT	0.043	0.027	0.106
FFP compared to	CFS	0.083	0.039	0.032
	6-min-WT	0.029	0.043	0.491
CFS compared to	6-min-WT	0.053	0.033	0.111

LFI, Liver Frailty Index; SPPB, Short Physical Performance-Battery; FFP, Fried phenotype; CFS, Clinical Frailty-Scale; 6-min-WT 6-min walk test

Supplementary Table S4. Estimated hazard ratio for 12-month mortality of patients diagnosed with physical frailty in univariate analysis and after adjustment for age, gender, and MELD score

	Univariate analysis		Multivariate analysis	p value
	HR (95% CI)	p value	HR (95% CI)	
Age (per year)	1.015 (0.976-1.055)	0.458		
Gender (Male)	0.913 (0.354-2.355)	0.850		
MELD score	8.168 (3.052-21.860)	<0.001	6.302 (2.087-19.033)*<br>6.788 (2.496-18.461)**<br>6.785 (2.502-18.402)***<br>8.168 (3.052-21.860)****<br>6.085 (2.182-16.968)*****	0.001*<br><0.001**<br><0.001***<br><0.001****<br>0.001*****
Liver Frailty Index	4.649 (1.479-14.608)	0.009	3.130 (0.966-10.140)	0.057
Short Physical Performance Battery	3.816 (1.505-9.676)	0.005	2.940 (1.141-7.572)	0.025
Fried phenotype**	4.270 (1.521-11.985)	0.006	3.001 (1.054-8.546)	0.040
Clinical Frailty-Scale***	5.020 (1.942-12.977)	0.001	NS	NS
6-min walk test***	4.566 (1.769-11.788)	0.002	2.783 (1.036-7.477)	0.042

Supplementary Table S5. Log Rank tests from Kaplan Meier survival curves for frail versus non-frail patients with 5 screening tools for frailty

Physical Frailty tool	3 months Long Rank p value	6 months Long Rank p value	12 months Long Rank p value	18 months Long Rank p value
Liver Frailty Index	0.004	0.008	0.004	<0.001
Short Physical Performance Battery	0.005	0.006	0.002	0.001
Fried Frailty Phenotype	0.002	0.002	0.003	<0.001
Clinical Frailty Scale	0.004	<0.001	<0.001	<0.001
6-Min Walk Test	0.007	0.009	0.001	<0.001

Supplementary Figure S1. Receiver operating characteristic (ROC) curve of the Liver Frailty Index measurement for differentiating survivors from non-survivors. The cut-off point of 4.34 yielded a sensitivity of 70% and specificity of 71.8%.

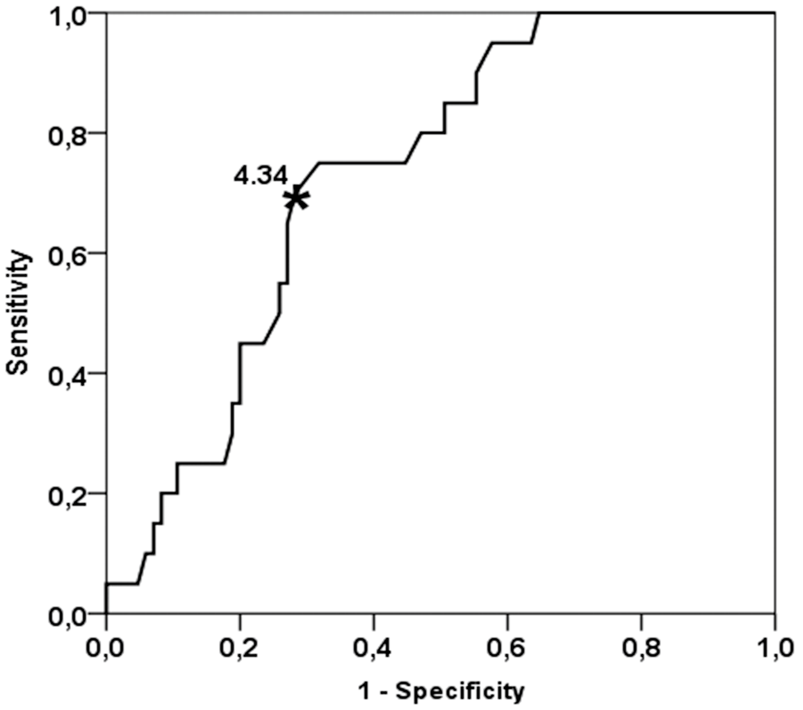

Supplementary Figure S2. Comparison of predictive ability of the AUROCs corresponding to Liver Frailty Index, Short Physical Performance Battery, Fried Frailty Phenotype, Clinical Frailty Scale, and 6-Min Walk Test for 18-month mortality

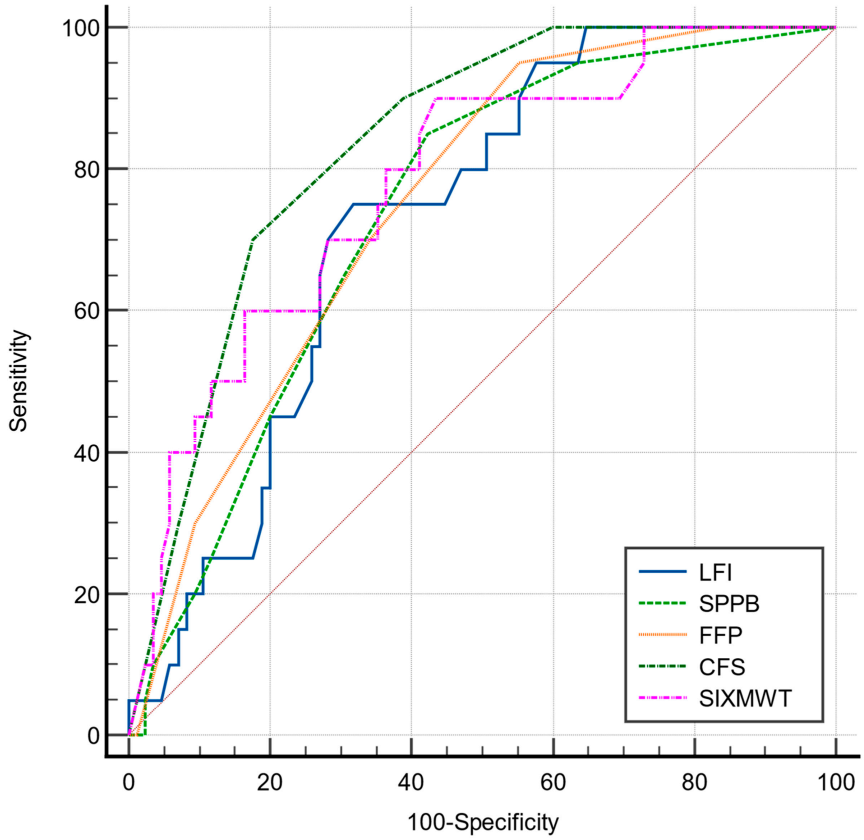

Supplement: Supplementary file 1 [file jcm-13-05169-s001.zip › jcm-3126550-supplementary.pdf]
